# From Knitting Technology to Robotics: Untethered Thermally Actuated Textile Exoskeleton for Dexterity Applications

**DOI:** 10.1002/advs.202509870

**Published:** 2025-08-11

**Authors:** Ibrahim Adel Khamis Ahmed, Munire Sibel Cetin, Kadir Ozlem, Asli Tuncay Atalay, Gökhan Ince, Ozgur Atalay

**Affiliations:** ^1^ Faculty of Textile Technologies and Design Textile Engineering Department Istanbul Technical University Istanbul 34437 Turkey; ^2^ Faculty of Computer and Informatics Engineering Computer Engineering Department Istanbul Technical University Istanbul 34469 Turkey

**Keywords:** 3D knitting, low‐boiling liquid, soft robotics, soft sensors, textile sensors, thermo‐active actuators, wearable robotics

## Abstract

Soft wearable robotic devices offer significant potential for human mobility assistance and rehabilitation; however, existing solutions are often hindered by bulkiness, limited scalability, and restricted portability. This study introduces a textile‐based exoskeleton glove equipped with thermally driven actuators, achieving dexterous motion in under 12 s using only 10.8 W of power while maintaining a low operating temperature of 48 °C. This performance surpasses the fastest previously reported system in terms of power input and operating temperature, which achieved actuation in 10 s but required 15 W and operated at 100 °C. In comparison, recent studies report response times of 120 s, with 14 W consumption and temperatures near 95 °C. The actuators utilize low‐boiling‐point liquids that undergo phase transitions upon heating, enabling fast, untethered actuation without external systems. The seamless knitted structure integrates sensing and actuation functionalities, including self‐return to initial position capability. This is achieved through digital machine knitting of specific patterns using functional yarns. The actuators demonstrate 270° bending, generating 2 N gripping force while maintaining energy consumption efficiency. The glove is mountable on an industrial robotic arm, demonstrating its ability to grasp and relocate objects. This study presents a quick‐response, scalable, energy‐efficient solution for wearable robotics.

## Graphical abstract

A textile‐based exoskeleton glove with integrated thermally driven actuators achieves rapid, low‐power response for dexterous motion. Utilizing advanced knitting methods, embedded heaters, and hybrid yarn sensors, the glove bends in a segmented fashion under 12 s at only 10.8 W and 48 °C. The system outperforms current state‐of‐the‐art soft actuators in efficiency, scalability, and wearable compatibility.

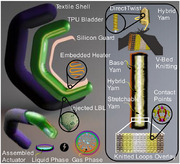

## 1 Introduction

The advancement of soft actuators using wearable textile materials represents a significant breakthrough in developing adaptable, lightweight, and flexible solutions for human mobility assistance.^[^ ^1^, ^2^ ^]^ These actuators are primarily constructed from specific textile materials, which are chosen for their lightweight and durable properties. Common fabrics include woven, knit, non‐woven textiles, and stretchable elastomers that can withstand repeated deformation.^[^ ^3^, ^4^ ^]^ By combining the mechanical properties of soft textiles with responsive actuation techniques, these systems are able to leverage advanced materials and fabric structures to achieve actuators that enhance comfort in wearable applications by providing effective breathability control and thermal regulation.^[^ ^5^, ^6^, ^7^, ^8^ ^]^ Their applications span from rehabilitation and assistive robotics to smart wearable technologies. Unlike conventional rigid actuators, textile‐based soft actuators provide superior comfort, adaptability, and seamless integration with the human body.

There are several innovative methods utilized in soft actuation, including air, pneumatic, and Low Boiling Liquid (LBL) of which the pneumatic technology is the most common. Air system isutilized in the case of prioritizing response time; However, this system requires rigid components including pumps, valves and connection tubes, which degrade the nature of the system to be applicable to soft wearable robotics; Therefore, LBL is an attempt to preserve this nature by eliminating any of these rigid components, furthermore, this study presents an integrated system where actuation is combined with the ability to return to initial shape while providing a sensor signal indicating the position, all compacted in one untethered form that can be easily scaled and mass produced due to the utilization of seamless knitting technology.^[^ ^9^, ^10^ ^]^ In this method, soft actuators are activated through the pneumatic pressurization of a bladder made from air impermeable materials like elastomers or thermoplastics.^[^ ^11^, ^12^, ^13^ ^]^ These materials are chosen for their high elasticity and durability, enabling the bladder to expand and contract effectively under varying pressures. Thermoplastic PolyUrethane (TPU) is highlighted for its ability to be customized in terms of hardness and elasticity, making it suitable for various soft robotics applications.^[^ ^14^, ^15^, ^16^, ^17^, ^18^, ^19^, ^20^, ^21^ ^]^ The plasticization of TPU enhances its flexibility, enabling the creation of actuators that can perform intricate movements. Additionally, other potential materials that can be integrated with TPU to enhance performance such as conductive materials for sensing capabilities and reinforcement fibers for improved structural integrity.^[^ ^22^, ^23^, ^24^ ^]^ However, pneumatic actuation technology has some drawbacks when used outside clinical settings, particularly for continuous at‐home assistance and rehabilitation during daily activities, precisely where such support is most needed, this is due to its reliance on external components such as compressors, valves, and connecting tubes, which add bulk and limit mobility.

To overcome these challenges, alternative actuation methods had to be explored.

Recently, Thermally Poured Soft Fluidic (TPSF) actuation has emerged as a promising alternative to pneumatic systems.^[^ ^25^, ^26^, ^27^, ^28^ ^]^ In this approach, actuator pressurization is achieved through the phase‐change of an injected liquid into vapor, eliminating the need for these limiting external components.^[^ ^21^, ^29^ ^]^ The choice of liquid is essential for achieving efficient actuation, as the liquid must evaporate quickly upon heating and condense effectively when cooled; Therefore, LBL plays a critical role in the actuator's functionality, as it facilitates rapid phase transitions that generate movement. By leveraging the unique characteristics of these liquids, the actuators can achieve significant displacement and responsiveness with relatively low energy input. This results in an entirely untethered actuation system that operates independently, requiring only an external electrical power source, an aspect that can be easily addressed using a portable power bank.

Although pneumatic systems suffer from above mentioned limitations, it excels in minimizing the actuation response time. Experimental characterization and modeling of several soft air bladders, focusing on its actuator performance and response time, particularly in applications that require precision and manual dexterity, achieving actuation within milliseconds.^[^ ^30^, ^31^, ^32^ ^]^

Focusing on soft actuators driven by LBL, this study presents an exoskeleton glove equipped with textile‐based actuators that integrate self‐actuation, sensing, and a shape‐memory‐like capability. Designed for high power efficiency, this system paves the way for fully untethered applications. These actuators harness the two‐way phase‐change behavior, wherein a liquid rapidly evaporates within 7 s upon modest thermal input, producing expansion and mechanical motion. This mechanism enables reversible deformation upon vapor condensation in a period of 42 s, resulting in precise and dynamic actuation cycles.

Furthermore, incorporating these systems into textile substrates allows for versatile shaping by leveraging the interchangeable properties of various yarns and fabrics. This design flexibility enables the deformable of the pouches into a wide range of angles, providing the necessary range of motion for different limbs or joints, enhancing the user's comfort and functionality in adaptive assistive technologies.^[^ ^30^, ^33^, ^34^ ^]^

Unlike traditional rigid sensors, soft robotic systems require sensing mechanisms that are lightweight, flexible, stretchable, and capable of conforming to complex, dynamic surfaces. These sensing systems are fundamental for enabling precise motion control, feedback mechanisms, safety responses, and adaptive behavior, all of which are essential for soft robotic devices operating in unstructured or human‐interactive environments.

Textiles, with their inherent flexibility and scalability, serve as an ideal platform for embedding such sensing systems. By leveraging advanced fabrication techniques such as knitting, weaving, and embroidery, various conductive and hybrid yarns can be integrated directly into the fabric structure to form strain sensors, pressure sensors, temperature sensors, and more. These textile‐based sensors are capable of detecting subtle deformations, stretches, and compressions in the fabric, allowing soft robots to sense their own movements and external stimuli in real time.^[^ ^35^, ^36^, ^37^, ^38^, ^39^ ^]^

Furthermore, the adaptability of textile manufacturing enables custom sensor geometries and distributed sensor networks across large surface areas without compromising softness or comfort. Conductive threads, often composed of materials like silver‐coated yarn or carbon composites, can be seamlessly interwoven to serve both structural and electrical functions, minimizing bulk and enhancing integration. In applications such as wearable exoskeletons, rehabilitation gloves, and robotic skins, textile‐based sensors provide critical real‐time feedback on bending angles, pressure distribution, and contact force enhancing the robot's responsiveness and enabling safer, more intelligent interaction with humans.

In summary, smart textiles are not merely enhancing existing products; they are redefining what is possible in soft robotics by enabling the creation of fully integrated, highly responsive, and human‐compatible sensing systems.^[^ ^40^ ^]^

The compatibility of the mentioned soft materials has facilitated the design, modeling, and assessment of fabric‐based actuators for wearable assistive gloves. Experimental evaluations confirm their effectiveness in improving hand mobility and functionality. Engineered to replicate natural hand movements, these actuators enhance tasks such as gripping and pinching. Additionally, the development of mathematical models for textile‐based thermal actuators in soft robotics applications enables precise control over hand motions, further optimizing their performance.^[^ ^31^, ^41^ ^]^

When analyzing the shape and movement of fingers, the accumulation of skin creases on top of each knuckle is prevalent, while a concentric bending between the links is sufficient in rigged actuators, it is not in the case of soft actuators, each joint requires additional regional stretching to create a 90° angle in order to simulate the humanoid knuckle movements.

Several studies have explored similar concepts in soft robotics using advanced knitting techniques.​ For instance, researchers have utilized 3D weft knitting to create small‐scale pneumatic actuators embedded within textiles. These actuators demonstrate tunable mechanical properties and can be integrated into wearable devices.^[^ ^42^ ^]^

Additionally, the PneuAct project from MIT introduced a method for digitally fabricating soft pneumatic actuators via machine knitting. This approach allows for the integration of sensing capabilities and the programming of actuator bending through elastic stitches​.^[^ ^43^ ^]^

Furthermore, the concept of PneumaKnit involves actuated architectures achieved through wale‐ and course‐wise tubular knit‐constrained pneumatic systems. This method explores the potential of morphable soft robotic systems by utilizing continuous variation in stitch structures.^[^ ^44^ ^]^

**Figure 1:**
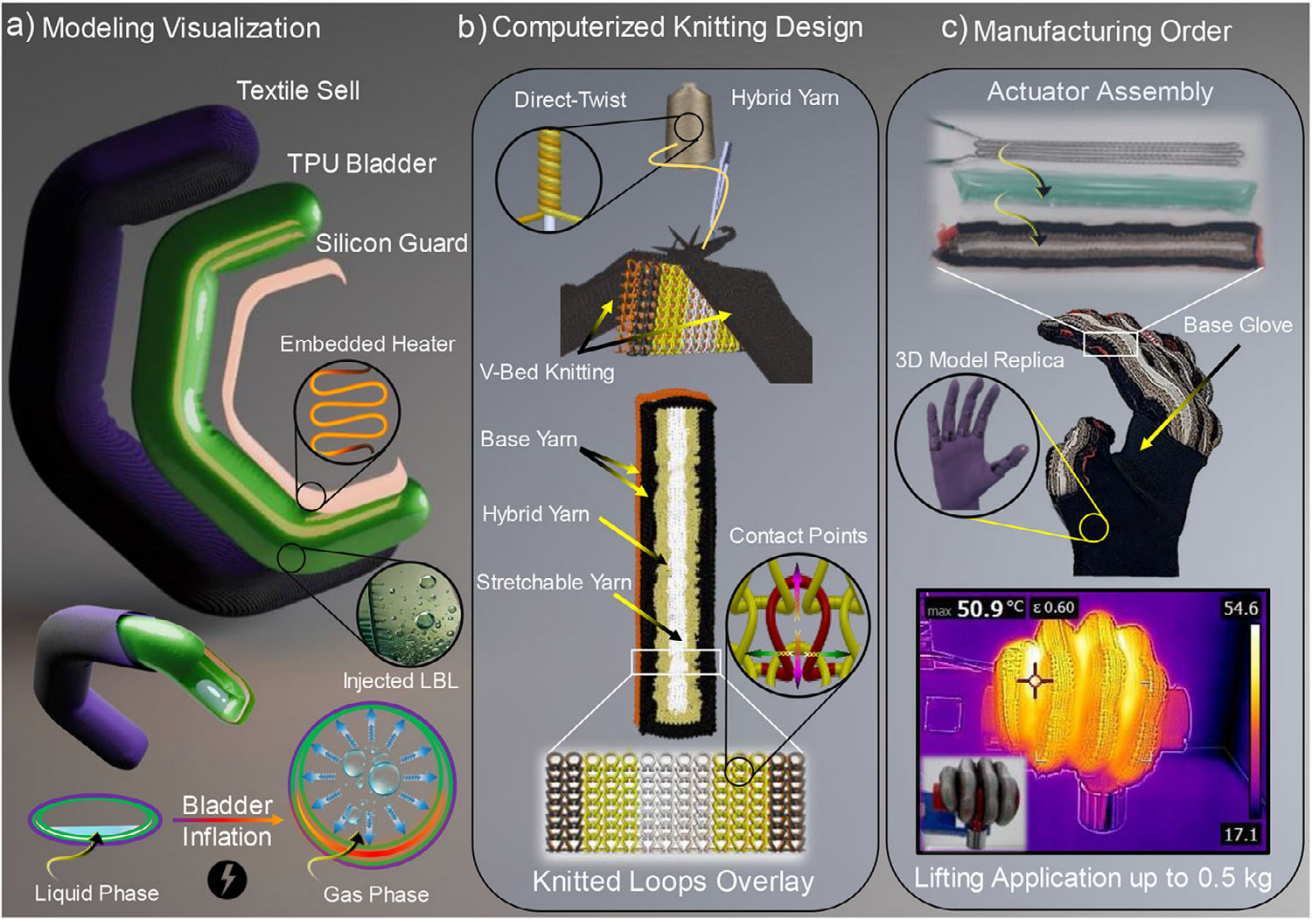
a) Modeled actuator working principle with the bladder injected with LBL and the integrated silicon guard impeded within the thread heater. b) Visualization of the knitting actions through computer design at the top, virtual sample's loops overlay with the loop's contact points at the bottom. c) Manufacturing order of the actuators constructing the exoskeleton glove at the top, with thermal imaging demonstrating the lifting capability application at the bottom.

The fabrication of the proposed integrated system employed these advanced materials with innovative methods illustrated in **Figure** **1**a.

The textile actuator shell is designed using a combination of four different yarns, each chosen for its specific properties, such as yarn count, stretchability, and electrical conductivity. Flat knitting technology is used for its versatility in producing complex knitting patterns, as shown in Figure 1b. This method allows seamless integration of the yarns into a stable knitted fabric, with each yarn knitted in a specific pattern to serve its intended purpose. The knitting patterns support the necessary actuator movements. Additionally, a stretchable conductive hybrid yarn is incorporated to improve the performance of the integrated strain sensor and ensure the system's scalability. The use of stretchable conductive hybrid yarn is a pivotal innovation in the development of textile‐based strain sensors, as it provides a harmonious blend of electrical conductivity, mechanical flexibility, and durability, all essential for integration into soft robotic systems and wearable electronics.^[^ ^45^ ^]^ In this study, the hybrid yarn is engineered through a direct twisting method, wrapping a silver‐coated conductive yarn around a spandex elastomeric core.

The selected LBL has an ambient temperature boiling point to ensure compatibility with human applications. The bladder size and LBL volume are optimized to minimize actuation time and improve electrical power efficiency. The integrated heater uses a separate conductive thread for thermal heating, paired with a rubber‐based silicone guard. This unique design promotes even heat distribution while protecting against excessive temperatures. The entire system is easily reproduced at various scales to match different finger lengths and knuckle positions. Consequently, an exoskeleton glove is developed to fit a 3D‐printed hand model, which is mounted on a robotic arm to demonstrate its ability to grasp and relocate objects of different shapes and weights, as shown in Figure 1c.

**Table 1:** Brief compilation of LBL soft actuators characteristics compared to the presented study.

No.	Article	Liquid	Volume [mL^3^]	Bladder	Size [mm * mm * mm]	Shape	Heating Source	Time [s]	Pressure [kPa]	Force [N]	Temperature [°C]	Power [W]
1	Akagi et al. (2015)^[^ ^22^ ^]^	Novec 7000	50	Plastic Laminates	110 * 90 * 6	Envelope	Integrated	35	N / A	N / A	250	40
2	Sanchez et al. (2020)^[^ ^25^ ^]^	Novec 7000	1.5	TPU Film	45 * 45	Pouch	Integrated	30	80	N / A	80	3
3	Usui et al. (2016)^[^ ^26^ ^]^	Novec 7000	0.016	Nylon Film	10 * 10	Pouch	Integrated	400	14	3.7	49	0.3
4	Narumi et al. (2020)^[^ ^46^ ^]^	Novec 7000	0.57	Aluminum	25 * 100	Pouch	Integrated	10	N / A	12	100	15
5	Uramune et al. (2021)^[^ ^47^ ^]^	Novec 7000	0.5	Nylon Film	40 * 40	Pouch	Separate	90	130	15.5	42	N / A
6	Atalay et al. (2024)^[^ ^40^ ^]^	Novec 7100	2	Latex Membrane	225 π * 120	Chamber	Integrated	60	107	0.005	85	15
7	Celebi et al. (2022)^[^ ^41^ ^]^	Novec 7000/7100	0.6	PA/PE Film	30 * 115	Pouch	Integrated	300	80	N / A	44	9
8	Taherkhani et al. (2024)^[^ ^48^ ^]^	Acetone	N / A	EcoFlex Mold	N / A	Multi Chambered	Integrated	120	N / A	N / A	60	15
9	Han et al. (2019)^[^ ^49^ ^]^	Ethanol	0.5	EcoFlex Mold	8 π * 58	Multi Chambered	Separate	55	N / A	14.5	140	N / A
10	Presented Study	Novec 7000	0.2	TPU Film	15 * 120	Pouch	Integrated	12	109	2	48	10.8

## 2 Results and Discussions

**Figure 2:**
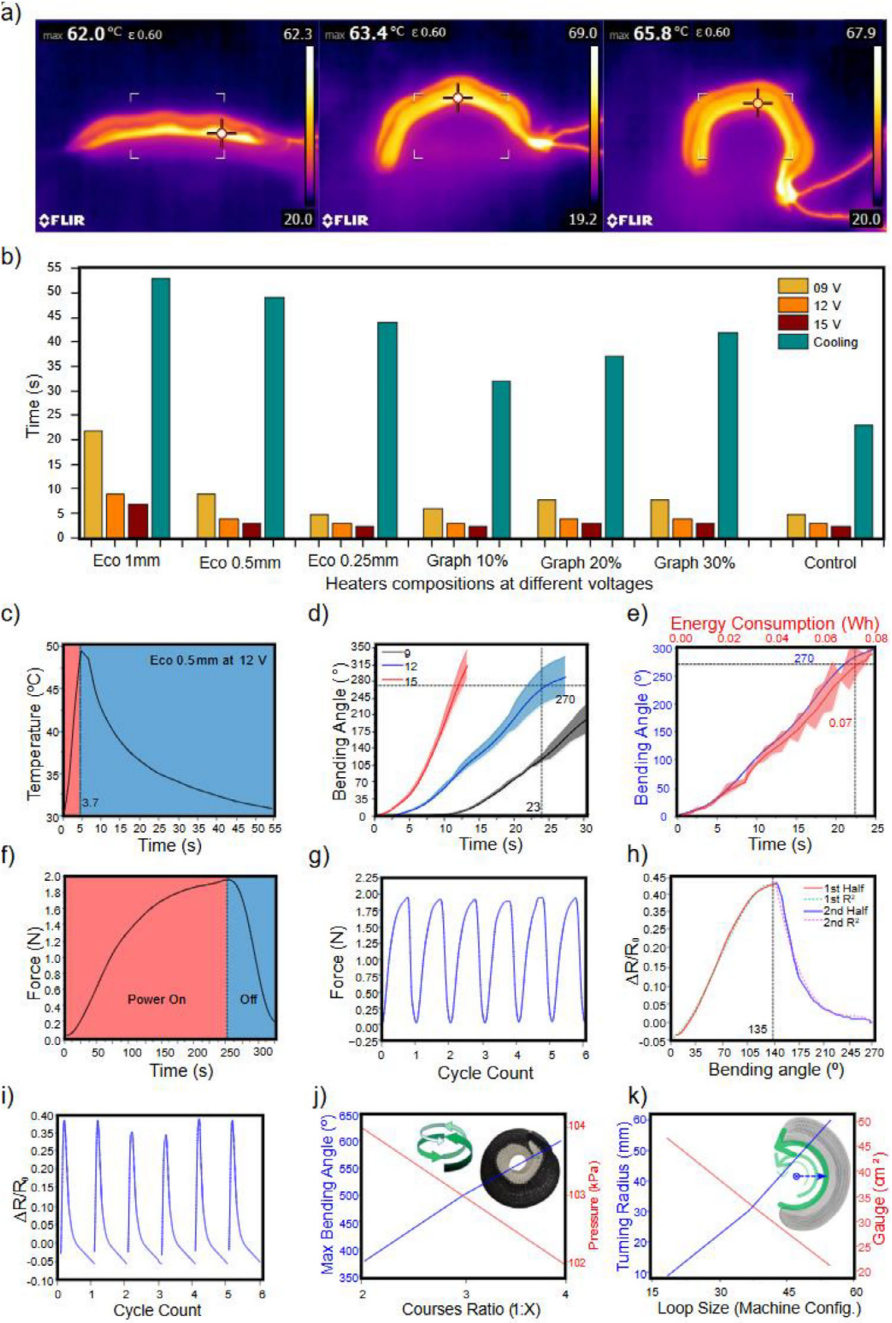
a) Thermal imaging of the actuator as it bends in a segmented manner. b) Comparison study for the heating and cooling performance of different silicon guard's compositions. c) Integrated heater required times for heating and cooling. d) Actuator bending angle changes over time. e) Energy consumption related to the change of bending angle over time. f) Generated grip force over time in a low power cycle. g) Grip force cyclic test. h) Measurements of relative resistance over the bending angle. i) Relative resistance changes cyclic test. j) Maximum bending angle and the required pressure in relation with the course ratio between the front and back actuator's sides. k) Turning radius and knitted fabric gauge relation with the loop size for the standard actuator length of 15 cm.

Experimental evaluations demonstrate that the actuator exhibits rapid response characteristics, achieving full bladder expansion approximately within 7 s under optimal conditions shown in Video S1 (Supporting Information). Following this initial expansion, with additional heating a subsequent increase in internal pressure facilitates controlled flexion and bending of the actuator. This results in a segmented actuation pattern that replicates the three bending angles of a human finger, forming three consecutive right angles, **Figure** **2**a presents the surface temperature of the textile actuator encapsulating the bladder with the integrated Stainless‐Steel Threads (SST) heater, captured via thermal imaging. The experiment is conducted at 22 °C (room temperature), and the power supply is turned off upon reaching a 270° bending angle to prevent any overheating or excessive stretching of the TPU.

To evaluate the effect of varying power inputs on the heating time, three different voltages 9, 12, and 15 V are applied to a control sample of the heating element. The corresponding times required to reach a target temperature of 50 °C are recorded as 5, 3, and 2.5 s, respectively. This control sample consisted of SST arranged in a serpentine pattern and secured solely with electrical tape. However, this configuration is susceptible to mechanical deformation, such as twisting or bending, which could lead to unintended electrical connections, resulting in uneven heating and localized hot spots, thus the need of silicon guard is realized.

The effect of adding silicone guard on the heating and cooling times is investigated by measuring the time required to reach 50 °C and cool back down to 30 °C. For silicone guard thicknesses of 0.25 and 0.5 mm, the effect is negligible, with heating times differing by only 1 s when 12 and 15 V power inputs are applied, resulting in total heating times of 3 and 4 s, respectively. Further efforts to reduce response time involved incorporating graphite into the silicone mixture at weight percentages of 10, 20, and 30% aiming to enhance thermal conductivity and power efficiency, where the time saved to reach the targeted temperature is only 1s as illustrated in Figure 2b.

Based on the analysis of the effects of different power inputs and silicone guard compositions, a 12 V supply with a plain 0.5 mm silicon guard is selected due to its ease of fabrication and operation. Figure 2c illustrates the heating and cooling profile of the selected silicone guard as it requires 3.7 s to reach 50 °C from an initial temperature of 30 °C and ≈52 s to cool back down. The marginal time savings from additional processing steps are deemed insufficient to justify the extra effort. Experimental testing of the knitted actuators revealed that it require ≈12 s to achieve a bending angle of 270° for the standard actuator length of 15 cm as illustrated in Figure 2d. Continued heating beyond this point resulted in additional bending, exceeding the initially targeted angle.

The energy consumption of the actuator is calculated based on the selected power supply and the time required to reach the target bending angle illustrated in Figure 2e. Over the 23 s heating period, the total energy consumption is determined to be 0.07 Wh. Compared to the previously designed actuator,^[^ ^40^ ^]^ this new design demonstrated significantly higher energy efficiency while achieving twice the bending angle, attributed to the use of a lower‐resistance heating thread with improved heat transmission.

The key factors for achieving higher power efficiency with quicker response times stem from the structural design of the proposed actuator, in the presented research, unlike previous studies, the engineered heating element is to effectively transfer heat, and also precise LBL volume is optimized to the intended application. Although Novec 7000 boils near ambient temperature, many of the previous studies relied on external heating sources or didn't prioritize the thermal transfer through adequate contact with their LBL, which exhibits low thermal efficiency. In contrast, this study utilizes an integrated heating element placed in close proximity to the LBL,^[^ ^26^, ^41^, ^47^, ^48^ ^]^ separated only by a 200‐micron‐thick TPU film. This in‐near‐direct‐contact heating element significantly enhances heat transfer efficiency. It is also fair to mention that these efficiencies are difficult to accurately measure and compare due to the different applications set ups utilizing different heat transfer methods, never the less, it is evident from the declared results that thermal conduction through a very thin film is far superior to other methods as listed in Table. Additionally, optimizing the volume of LBL for the specific application minimizes unnecessary heat input by reducing the energy needed for latent heat transition, further improving overall system performance. It is important to note that not all of these studies tested their actuators under optimal or standardized conditions. As a result, comparing them based on power input relative to response time may not be entirely appropriate, especially in cases where the target applications did not require high force output.(Table **1**)

During the gripping force test, the actuator generated a force of 2 N while being gradually heated over a period of 4 min, maintaining a temperature below 50 °C illustrated in Figure 2f. Following this, the actuator is powered off for 1 min to allow complete cooling to room temperature before repeating the heating cycle. This process is conducted for a total of 6 cycles to evaluate the actuator's reliability under repeated use illustrated in Figure 2g.

The extended actuation time observed under lower power inputs is attributed to the vertical orientation of the actuator during testing. In this setup, the LBL accumulated at the bottom rather than distributing evenly across the heated area, as it would in a horizontal configuration, electing so to mitigate potential heat damage associated with higher power inputs.

It is important to note that during the gripping application test, the second heating cycle is significantly shorter than the first. This is because the initial cycle requires priming the heater from room temperature (22 °C) up to ≈33 °C, just below the boiling point of the LBL. Once this baseline temperature is reached, subsequent cycles require only a few seconds to generate the same force, enabling rapid and efficient repeated actuation.

The results of the yarn‐based knitted resistive sensor are consistent with previous studies,^[^ ^50^, ^51^ ^]^ demonstrating a linear relationship R^2^ equal to 0.97 from 0°:140° and R^2^ equal to 0.92 from 140°:270° between resistance change and bending angle up to the maximum stretch of the knitted loops in the course direction. Beyond this point, further stretching occurred in the wale direction, causing the conductive yarn fibers to cluster more tightly, leading to a progressive decrease in resistance as illustrated in Figure 2h.

The decrease in resistive sensor values during actuation is also influenced indirectly by rising temperatures, as increased thermal energy excites electrons, enhancing conductivity. To evaluate this effect, a dedicated test was performed on a stationary actuator heated to 50 °C, as shown in Figure S1 (Supporting Information). Results indicate that the temperature‐induced resistance drop is relatively minor, accounting for ≈10% of the total resistance variation observed during actuation. In future work, this thermal influence can be mitigated by integrating a non‐stretchable conductive yarn from the same type added to the border of the textile shell, acting as a temperature sensor. Any increase in conductivity from this element could be monitored and compensated for by the control system.

The integrated U‐shaped resistive sensor exhibited an initial resistance of 40 Ω before actuation, increasing linearly to 54 Ω at a bending angle of 140° with a standard deviation of 0.5. Beyond this angle, a gradual decrease in resistance is observed, eventually returning to its initial value at 240° and down to 38 Ω at the targeted bending angle of 270° with a standard deviation of 0.35, these differences are corresponding to a change of 0.35 upward the midpoint of the cycle and the other half return change is 0.3.

During sensor testing, it is noted that resistance changes are more gradual during the longer cooling phase as the actuator returns to its original shape, allowing for more precise readings. Based on this observation, the test is repeated six times, starting from the target bending angle and moving backward to the initial position. The results demonstrated consistent resistance readings across cycles, with no signs of degradation, confirming the superior performance of hybrid yarn compared to purely conductive yarn in maintaining sensor reliability and accuracy illustrated in Figure 2i.

Observing the symmetry of the resistance graph around ≈140° bending angle, it becomes evident that the two bending phases can be easily distinguished. This distinction can be made by simply verifying whether the power is on or off during the resistance change, allowing for accurate identification of the actuator's movement direction.

Building upon previous designs, which enables fluidic circular motion, this work introduces an advanced iteration by applying regional loop accumulation, a technique also known as pintuck knitting to achieve the targeted segmented bending. The accumulation ratio is designed such that two rows of knitting are applied to the upper faces of the actuator structures, corresponding to the skin creases, while one row of knitting is applied to the lower faces (knitting row ratio 2:1), such ratio's effect on bending is illustrated in Figure 2j for the standard actuator length of 15 cm. In the regions corresponding to the flat sections of the finger, a 1:1 ratio is applied on both the upper and lower faces to prevent any unnecessary bending. Further fine‐tuning of the actuator's bending profile requires controlling its turning radius. This can be accomplished by adjusting the knitted loop size, which directly influences the fabric gauge. Smaller loops create a denser fabric, resulting in a smaller turning radius and, consequently, tighter bending, as demonstrated in Figure 2k.

All components used in this integrated system are commercially available and backed by high‐performance specifications. According to its technical data sheet, the TPU film (Stretchlon 200) has an elongation at break of 500%, a tensile strength of 55 MPa, and a maximum operating temperature of 121 °C. Similarly, the EcoFlex (00‐30) exhibits an elongation at break of 900%, a tensile strength of 1.3 MPa, and a maximum use temperature of 232 °C. The knitted shell, comparable to durable sportswear fabrics made from stretchable yarns, is designed to withstand daily use.

However, throughout this study's numerous trials, a few key factors were revealed that can impact actuator performance. Operating temperatures above 110 °C, particularly under a 15 V supply, led to TPU bladder degradation. 0.25 mm thickness silicone guards, challenging to mold precisely with laboratory equipment, often failed to maintain the serpentine heater pattern, causing short circuits and generated focused heated spots. Contact between the knitted shell and sharp objects, such as rough surface finish from the 3D‐printed parts or edges of objects being grasped, sometimes snatched the stretched loops, compromising structural integrity and actuation behavior. Additionally, Insufficient air removal before bladder sealing affected the condensation rate of Novec 7000, resulting in longer cooling duration and in extreme cases, the bladder's inability to deflate completely preventing the actuator from returning to its initial position. When these factors are carefully managed and specimens are properly fabricated and stored in cool conditions, the actuators operated cautiously retain stable performance for multiple months without degradation.

## 3 Application

The fabricated actuator is replicated five times, each varying in size and length to fit the corresponding fingers of a 3D‐modeled hand. These actuators are stitched onto a basic glove, which is then mounted onto the model hand. An illustration to the glove assembly is shown in Figure S2 (Supporting Information), showcasing the fitting process over a 3D printed hand model replica, this fitting required the actuators to be positioned precisely over the 3D model knuckles for a homogenous bending actuation, simulating a real case scenario.

**Figure 3:**
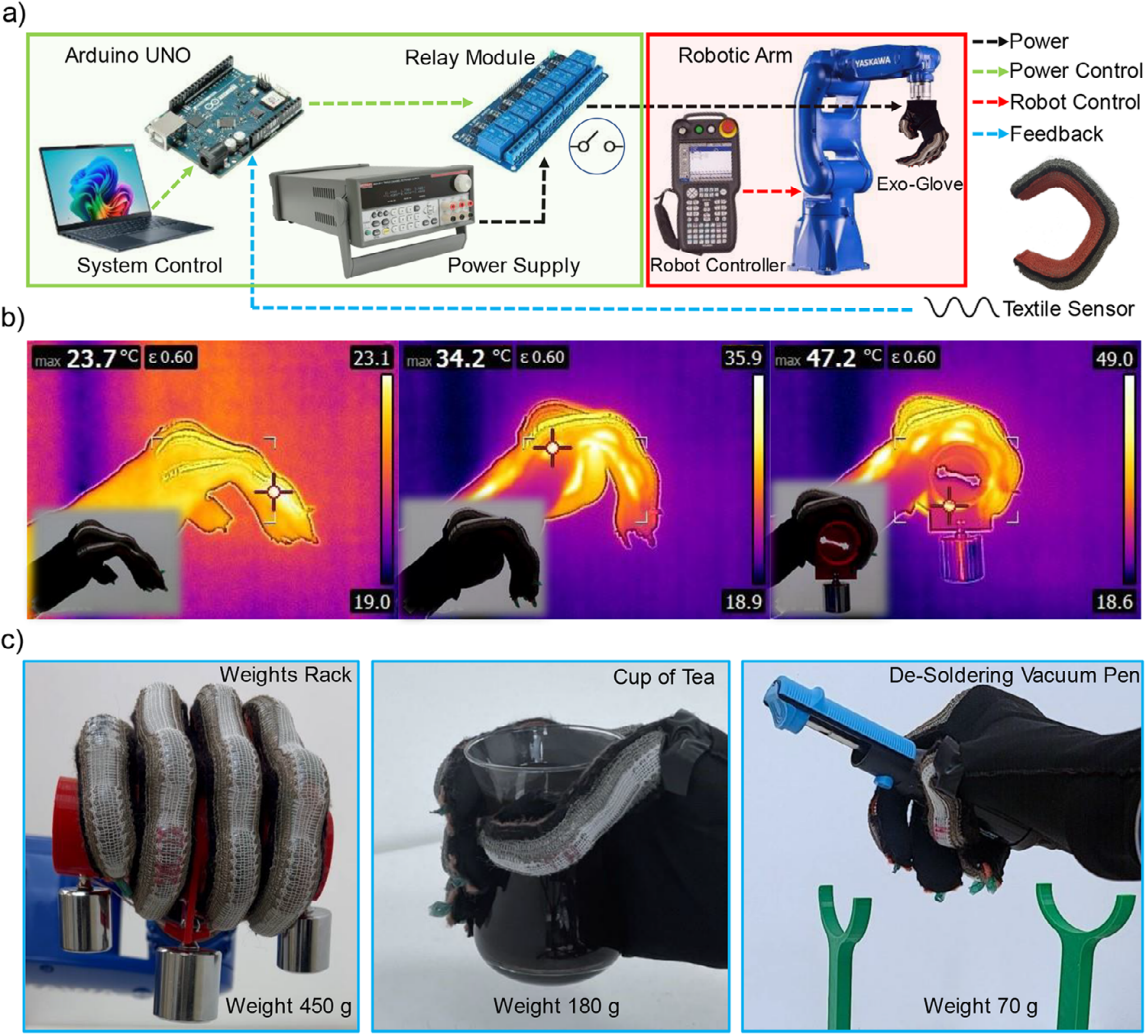
a) Diagram of the control systems operating the glove. b) Thermal imaging of the glove as it grasps. c) Glove applications of lifting and relocating different objects varying in shapes and weights.

When activated, the exoskeleton glove facilitated extension and flexion movements while being fixed to an industrial robotic arm. **Figure** **3**a illustrates the control system developed for the closed loop system utilized in the operation.

A thermal imaging showed the stages of finger bending of which the glove grasped a weight rack as shown in Figure 3b.

The proposed integrated system effectively demonstrated its ability to grasp, lift, and relocate objects of different shapes and weights, as depicted in Figure 3c. Ensuring that the actuator's inner diameter matched the circumference of the object allowed for full contact and secure handling. The glove successfully grasped a de‐soldering vacuum pen and relocated it to a tool bin, as shown in Video S3 (Supporting Information). Additionally, the actuator is tested for its lifting capability, successfully handling incremental weights up to 450 g, highlighting its capability to lift significantly heavier objects.

## 4 Conclusion

This study presents a novel approach to soft wearable robotics by integrating thermally actuated textile‐based actuators into an exoskeleton glove. The proposed system overcomes the limitations of conventional actuation methods by utilizing low‐boiling‐point liquids for rapid and energy‐efficient phase‐change‐driven motion. The seamless integration of sensing, heating, and actuation in a textile‐based structure enables a fully untethered and scalable solution.

The experimental results demonstrate the glove's capability to generate precise and dynamic actuation, achieving a 270° bending angle within 12 s while maintaining high energy efficiency. The actuators can operate at a voltage as low as 6 V with a low power consumption of 3.6 W, delivering a gripping force of 2 N. The system's performance is validated experimentally, where the glove successfully grasped and relocated objects of varying shapes and weights. Additionally, the integrated resistive textile sensors provided real‐time feedback, enhancing control and adaptability for dexterity‐focused applications.

This study underscores the advancements in LBL‐driven textile‐based actuators, particularly emphasizing several key innovations. Integrating a stretchable heating element directly into the bladder enables faster actuation with lower energy consumption. The use of diverse yarns with tailored mechanical and electrical properties supports a stable, segmented bending motion, made possible through advanced knitting techniques. Additionally, a hybrid yarn‐based resistive sensor is seamlessly embedded into the structure, delivering accurate and consistent readings over a wide range of deformation. Material optimization across the system significantly reduces the volume of LBL needed to achieve effective actuation. Altogether, these enhancements contribute to a highly flexible and responsive design, making the actuator well‐suited for human‐interactive applications. The result is a lightweight, adaptable, and customizable alternative to conventional rigid actuators, ideal for integration into next‐generation wearable and assistive technologies.

Future work will aim to further optimize actuator materials, improve system response time, and expand the range of real‐world applications, particularly in medical settings adding on a cloud‐based control system providing truly portable applications for at‐home therapeutical treatment.^[^ ^52^ ^]^ These continued advancements will contribute to the development of next‐generation soft robotic systems that emphasize efficiency, comfort, and functionality, pushing the boundaries of wearable assistive technologies.

## 5 Experimental Section

The design brings together textile‐based actuators with self‐heating TPU bladders that incorporate SST serving as resistive heating elements. Each bladder is injected with the corresponding LBL volume relative to its size. These bladders are embedded within the knitted glove fingers to initiate the bending. Upon applying electrical power to the SST, they generate heat and cause the LBL to evaporate, generating pressure that drives the actuator's movement. In this way, the pressure buildup in the bladder, enables the knitted fingers to bend, mimicking the natural movement of human fingers. Additionally, resistive textile sensors are integrated into the actuator fingers during the knitting process using conductive hybrid yarns. These sensors detect and measure bending motion, providing real‐time feedback for precision tasks.

The exoskeleton glove consists of three integrated components, with an external control circuit regulating power distribution. The specifications of each component are detailed in the following sections, arranged according to their fabrication sequence and illustrated in Video S2 (Supporting Information).

### Knitted Actuators

In this study, the purpose of the actuator's design is to mimic finger movement. The actuator shells are designed in a tubular shape with one open end to allow integration of the activation system, using only knitting technology without the use of any other textile production methods such as sewing, weaving etc., a monolithic structure where interconnected structural elements are made as a single piece. The designs are created using the Shima Seiki SDS‐ONE APEX4 pattern program, and the production is carried out using the Shima Seiki N.SVR 122 Computerized Jacquard Flat Knitting Machine (V‐bed; 14 gauge).

During the actuator design process, a U‐shaped resistive sensor is integrated in the upper surface of the actuators.

For the design to accommodate all fingers, it needs to have different sizes. Therefore, different repeated regions are added to the joint and flat regions of the finger. In this way, it is possible to produce different lengths for each area of ​​the finger. Taking the index finger as an example, from the fingertip to the palm, the finger consists of a total of 4 flat regions and 3 joint regions. Each region is allocated a repeat code in the patterned design, during production, the values assigned to these repeat codes can be altered on the machine allowing varying length productions.

In the finger‐shaped actuator design, to reduce the required activation time and support the targeted anisotropy, yarns with different elasticities are used regionally.

The yarn used on the lower surface of the actuator is (first yarn; 2/24 Nm Acrylic + Cotton). To enable the accumulation areas at the joints, a stretchable yarn (second yarn; Çombaş 140/70^*^2 Nylon + Spandex) is selected. For the sensor U‐shaped region, hybrid stretchable conductive yarns are produced on a DirecTwist 2C machine (third yarn; 140 den Spandex (core)), (fourth yarn; SHIELDEX 117/17 dtex HC +B 1‐ply silver‐coated polyamide (cover)) are used to match the stretchability of the second yarn allowing a homogeneous knitting.

Considering the overall design, the interlock technique is chosen, which allows multiple carriers to operate within the same row.

### Bladder—Specifications

The bladders are fabricated from TPU films (Stretchlon 200, Fiber Glast) to easily withstand the evaporation point of the 3M NOVEC 7000 of 34 °C and further heating up to 90 °C for faster actuation. The TPU film is cut into rectangles with the dimensions of 5 * **14 cm** and folded twice longitudinally into a **Z‐shape** to form two separate pockets. This configuration allowed for the encapsulation of the **LBL** within a fused chamber while positioning the **heating element** in the lower pocket to optimize heat transfer. Although maximum heat transfer would be achieved by fully submerging the heating element by the **LBL**, **i.e., one pocket**; unfortunately, this approach presented significant challenges, specifically, it is not feasible to securely fuse the TPU film around the electrical wires supplying power to the heating element, and there would be no forces applied onto the heating element to stretch and bend it along with the bladder. Consequently, the **final LBL chamber dimensions** are **1.5** * **12 cm**, forming a rectangular structure capable of inflating into a cylindrical shape with a volume of **8.6 cm^3^**. Defining the final shape and size of the bladder facilitated the calculation of the **LBL volume** required to achieve chamber pressurization, where the required liquid volume corresponding to different pressure values is determined using the general gas equation *PV = nRT*.

(1) $$n_{g}=\frac{P}{RT}\;*V_{g}$$

where *n_g_* represents the amount of gas in moles, *P* is the absolute gas pressure, *R* is the ideal gas constant, *T* is the absolute temperature, and *V_g_* is the gas volume. The mass (*m*) of the material in kilograms (kg) can be calculated using:

(2) $$m=\;Mn\;*\;n_{g}\;*\;10^{-3}$$

In this equation, M is the molar mass of the substance in grams per mole (g mol^−1^). For example, the molar mass of NOVEC 7000 is (200 g mol^−1^), and its density (*ρ*) is (1400 kg m^−^^3^).

To determine the required liquid volume (*V_l_*) for achieving the desired pressure, the maximum gas‐filled volume *V_g_* of the actuator is first calculated. For the rectangular thin‐film pouch used in bending actuators, the following equations apply:

(3) $$V_{l}=\frac{m}{\rho }\;$$

(4) $$V_{l}=\frac{Mn_{g}\;*\;\;10^{-3}}{\rho }\;$$

(5) $$V_{l}=\frac{m}{\rho }\;*\;\frac{P}{RT}*\;V_{g}*\;10^{-3}$$

Based on the general gas law, the calculated liquid volume required for complete evaporation and gas expansion within the actuator is ≈0.05 mL. However, this amount is only sufficient to erect the chamber to its cylindrical shape and does not generate the necessary bending force and the required grip force. To ensure adequate actuation, additional liquid must be injected to allow for bladder expansion, effectively doubling the initial volume, while also incorporating redundancy in case of an unaccounted losses during fabrication.

As the injected liquid volume increases, more heat is required for vaporization due to its higher heat capacity, causing a delay in the onset of inflation. Over time, pressure buildup raises the boiling point, leading to an excess of vapor inside the bladder. This results in a high condensation rate, which continues increasing until it reaches equilibrium with the boiling rate, at which point a pressure drop occurs.

In the experiment, an actuator containing 0.2 mL of liquid is activated, supplying 12 V at 0.9

### Bladder—Manufacturing

The rectangular sections of the TPU film are heat‐pressed at 120 °C, aligning the heating element to form the initial fold, thereby securing it within a secure pocket. This configuration allowed the heating element to stretch and bend as an integrated component of the bladder. The second fold is then fused using an impulse sealer (PCS 300, Brother) creating a pressure‐tight seal around the LBL chamber. Following sealing, the LBL is injected into its designated chamber, and a final fused seal is made after removing any trapped air. The completed bladder is subsequently tested through pressurization before insertion into the knitted actuator.

### Heater—Heating Element

SST (BEKINOX VN 12/2 ^*^ 275/175S, BEKINTEX) was utilized as the heating element due to their electrical resistance. Although the thread count is not suitable for direct integration with the knitted actuator, their resistivity and flexibility allowed for embedding within a silicone‐based elastomer to maintain a serpentine pattern. Each heating element incorporated a total thread length of 75 cm, resulting in a resistance of 13 Ω. Three voltage levels 9, 12, and 15 V are applied to the heating element to evaluate its suitability for fast‐response applications.

### Heater—Silicon Guard

EcoFlex (00‐30) silicone rubber is selected due to its low thermal conductivity and high stretchability. While the low thermal conductivity had a minimal impact on the actuator's response time at thicknesses of 0.25 and 0.5 mm, it facilitated a homogeneous distribution of heat from the embedded threads, preventing the formation of localized hot spots. Additionally, the material's high stretchability ensured that the heating threads maintained their serpentine pattern without unintended line connections, even as the heating element concaves in response to bladder pressurization and actuator bending. Since higher power levels had a negligible effect on the response time (only 1 s) while posing a risk of reaching temperatures that could damage the TPU films, their utilization is considered unnecessary.

### Heater—Manufacturing

A 3D‐printed mold with dimensions of 1 ^*^ 12 cm is designed with three different depths of 0.25, 0.5, and 1 mm. SST are arranged in six parallel lines before pouring 0.5, 1, and 2 g of an equal‐parts EcoFlex mixture corresponding to the mold's respective depth. The silicone is cured at room temperature for 2 h before integration into the bladder. Additionally, an experiment is conducted to enhance thermal conductivity by incorporating graphite into the silicone matrix as mentioned above.

### Control Circuit

An ARDUINO UNO is programmed for a basic ON / OFF control manner and connected to a five‐relay module (FL‐3FF‐S‐Z 5VDC). The relay module facilitated the electrical power transmission from the power supply to each actuator heating element, enabling active switching of the power supply via the computer‐controlled interface. The experimental analysis of the heating element's performance under varying power inputs determined that the maximum temperature achieved remained well below the melting point of the TPU film when supplied with 9 and 12 V. Consequently, the implementation of a closed‐loop control system with feedback signals is deemed unnecessary for lower power levels.

### Laboratory Characterization

All testing apparatus used in the characterization of this actuator are listed in the supporting information document where the setup configuration for each test is shown in Figure S3 (Supporting Information).

## Conflict of Interest

The authors declare no conflict of interest. The European Union is not responsible for the views and opinions expressed by the authors.

## Supporting information



Supporting Information

Supplemental Video 1

Supplemental Video 2

Supplemental Video 3

## Data Availability

The data that support the findings of this study are available from the corresponding author upon reasonable request.
