# Supplementary material for: From Knitting Technology to Robotics: Untethered Thermally Actuated Textile Exoskeleton for Dexterity Applications
Source: Adv Sci (Weinh). 2025 Aug 11;12(41):e09870. doi: 10.1002/advs.202509870 (PMC12591156; doi:10.1002/advs.202509870)
Supplement: Supplementary file 1 — Supporting Information [file ADVS-12-e09870-s004.pdf]

Supporting Information

From Knitting Technology to Robotics: Untethered Thermally Actuated Textile Exoskeleton for Dexterity Applications

*Ibrahim Adel Khamis Ahmed[1], Munire Sibel Cetin[1], Kadir Ozlem[2], Asli Tuncay Atalay[1], Gökhan Ince[2], and Ozgur Atalay[1, *]*

Abstract

This work presents a textile-based exoskeleton glove with integrated thermally driven actuators that deliver rapid, low-power, segmented bending motion for dexterous tasks. Combining advanced knitting techniques, stretchable heaters, and hybrid yarn sensors, the glove achieves up to 270° bending within 12 seconds at just 10.8 W and 48 °C. Optimized LBL phase-change actuation and embedded sensing ensure efficient, untethered performance. Demonstrated on a robotic arm, the glove reliably grasps and relocates varied objects, showcasing its potential for scalable, comfortable, next-generation wearable robotics.
The following are supporting information that aid in replicating the achieved results, signifying the design details and illustrating the characterization testing methods.

Components of Experiments

• *Thermal Camera (E5, Flir, Oregon, USA):* It is used for real-time observation of heat distribution and validation of surface temperature.
• *Digital Multimeter (34465A, Keysight, California, USA):* It is utilized for collecting resistance information from the resistive sensor.
• *Power Supply (2231A-30-3, Keithley Instruments, Ohio, USA):* It is used to transfer electrical energy to the heater created with the stainless-steel yarn.
• *Force Gauge (M5-50, Mark-10, New York, USA):* It is employed to obtain force information for cycle tests. Data was collected.
• *Universal Tensile Machine (ESM303, Mark-10, New York, USA):* It is used for positioning the actuator and force gauge during cycle tests.
• *Industrial Robotic Arm (GP8, Yaskawa, Slovenia):* The manufactured glove was mounted on it for demonstration of various themed applications.

Supporting Figures

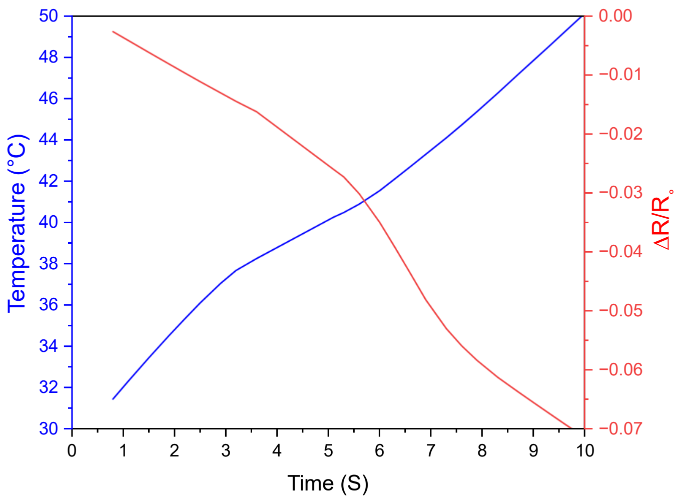

Figure S1: Influence of temperature increase on the textile resistive sensor while the actuator is stationary resulting in a minor decrease that could be mitigated.

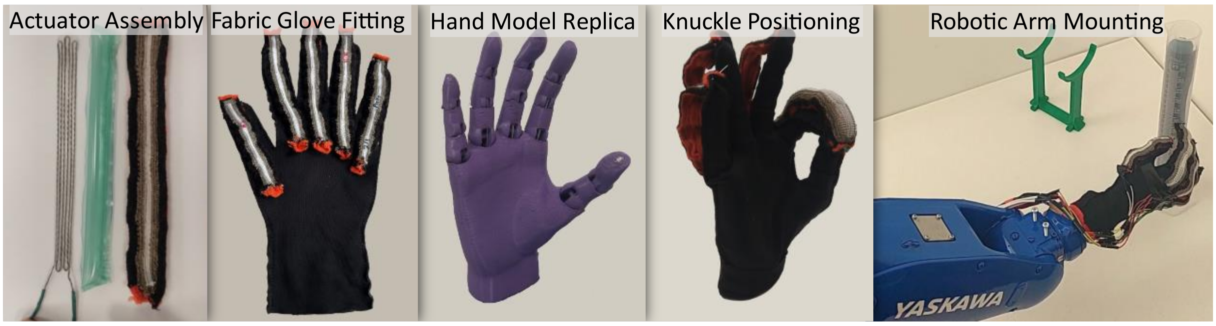

Figure S2: Illustration of all the components and their manufacturing order utilized in producing the wearable exoskeleton glove, simulating a real case scenario.

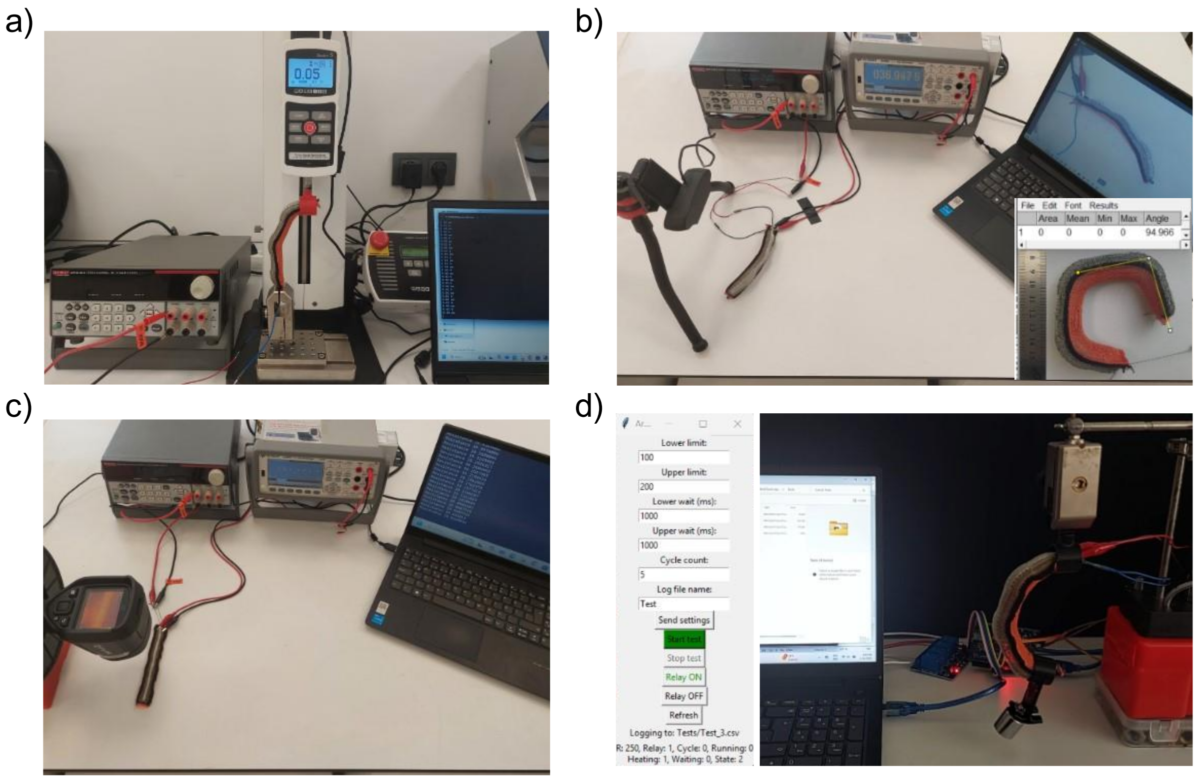

Figure S3: a) Grip force test setup where an actuator is fixed vertically to grip and pull down on the force gauge. b) Bending angle and resistance change with time testing setup where an actuator bending profile is recorded to be analyzed while the resistance changes are documented. c) Thermal imagery setup for the actuator components heating profile validation with resistance change documentation while inactive. d) Resistance changes while under load test setup with a closed loop control system active interface.

In this study an “ImageJ” tool was utilized in calculating the bending values where the activation of the actuator was recorded and the frames were latter analyzed to drive the plot.

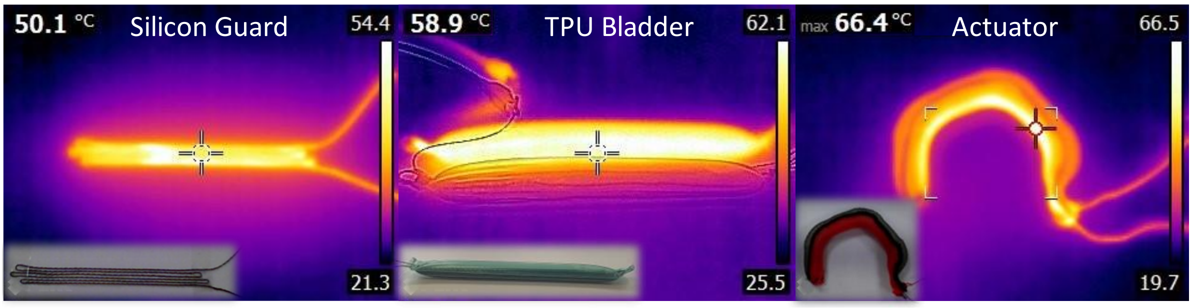

Figure S4: Thermal imagery of the actuator components at their activated timers where on the left is the heating silicon guard after nearly 4 seconds, in the middle is the TPU bladder full inflation after 7 seconds and on the right is the integrated actuator bending after 12 seconds.

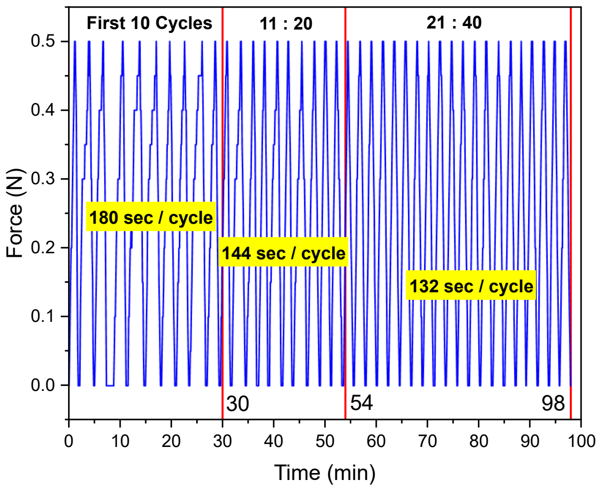

Figure S5: Grip force cycle test for durability validation where an actuator is subjected to 40 cycles of heating and cooling in a continuous session of 100 minutes.

Knowing that the research point of this study is towards wearable soft robotics intended for therapeutical assistance where the subscribed session hardly exceed few minutes, the results from this test demonstrates the actuator applicability in this filed with consistent performance, where the duration of the first 10 cycles required 2 minutes of heating and 1 minute of cooling, however the following 10 cycles showed a reduced heating duration due to the heat priming of the activation system, the completion of the priming showed further reduction in heat duration over the last 20 cycles, where the heating and cooling duration nearly matched at approximately 1 minute for each. After the completion of this test, the actuator was inspected and no signs of degradation was observed.

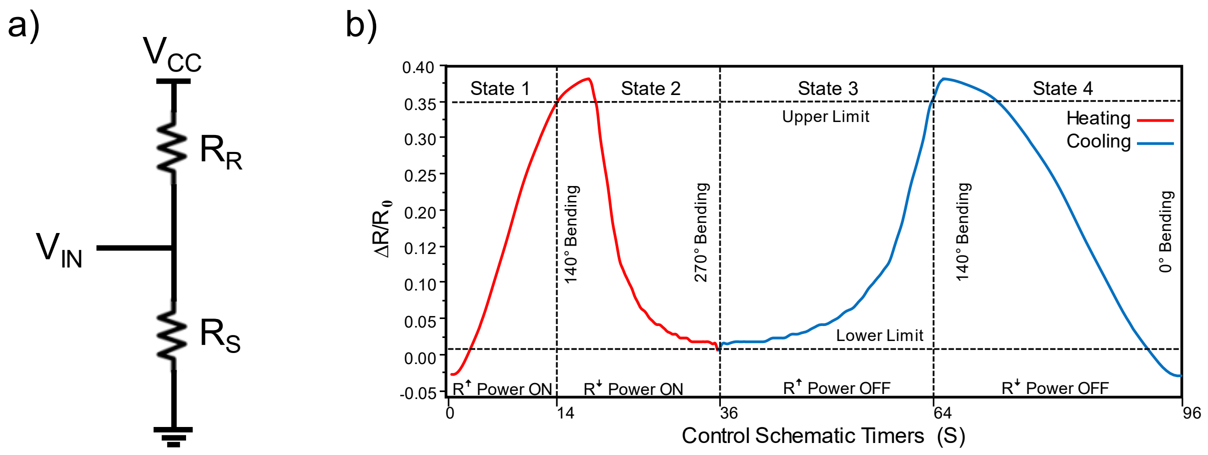

Figure S6: a) Closed loop voltage divider for sensor signal acquisition. b) Schematic plot of the four states which represent the sensor signal during full actuation from heating to cooling.

Figure S6(a) shows the schematic of the voltage divider circuit used for measuring the resistive sensor during the application test of the textile-based actuator. The voltage in the divider circuit is calculated using the following formula:

$$\frac{V_{IN}}{V_{CC}} = \frac{R_S}{R_R + R_S}, \tag{S1}$$

where V_{IN} is the input voltage measured by the Analog-to-Digital Converter (ADC) of the Arduino, V_{CC} is the supply voltage (fixed at 5 V), Rs is the resistance of the textile actuator's resistive sensor, and R_R is the fixed reference resistor. This equation is written in the form of Equation S2 and then rearranged into the formula presented in Equation S3 to compute R_S. In Equation S3, V_{CC} and R_R are constant values, and V_{IN} is obtained by measurement through the Arduino Uno; hence, all required variables are known. Using Equation S3, the resistance value of the sensor can be calculated.

$$V_{cc} * R_s = V_{in} * R_R * V_{in} * R_s \tag{S2}$$

$$R_s = \frac{V_{in} * R_R}{V_{cc} - V_{in}} \tag{S3}$$

As the actuator bends, the resistance of the sensor initially increases and then decreases. As illustrated in Figure S6(b), the sensor exhibits a symmetric behavior during both the bending and straightening phases. For the implementation, upper and lower resistance thresholds are defined for the increasing and decreasing phases of the sensor signal, and four different states are defined within the microcontroller accordingly. In State 1, the actuator is heated, and bending begins. When the resistance reaches the upper threshold, the system transitions to State 2. When the lower resistance threshold is reached, the bending phase is considered complete, and the power is cut. During natural cooling, the resistance first increases and then decreases; this phase is referred to as State 3. Once the resistance again reaches the upper threshold, the system enters State 4, during which the resistance briefly increases and then begins to decrease. When the resistance value drops to the lower threshold, one full cycle is completed. When a new bending command is issued, the actuator returns to State 1 and the cycle starts over.

The heating of the actuator is controlled via a relay based on feedback from the resistive sensors, and the entire cycle is managed by the microcontroller Figure 3(a). The recording of the sensor data and the issuance of new bending commands are handled by a Graphical User Interface (GUI) developed on a computer.

Technical Data

Table S1: Data sheet for Ecoflex™ detailing its physical and mechanical characteristics.

Shore Hardness	00-30
Specific Gravity	1.07 g/cc
Specific Volume	26.0 cu. in./lb.
100% Modulus	10 psi
Tensile Strength	200 psi
Elongation at Break	900 %
Die B Tear Strength	38 pli
Shrinkage	<.001 in. / in.
Mixed Viscosity	3,000 cps
Useful Temperature (min)	-65 °F
Useful Temperature (max)	450 °F
Pot Life	45 minutes
Cure Time	4 hours
Color	Translucent
Mix Ratio by Volume	1A:1B
Mix Ratio by Weight	1A:1B

Calculated Liquid Volume Modeling Equation

$$n_g = \frac{PV_g}{RT}$$

Where:

- Pressure P = 102.5 bar = 102.5 * 10^5 Pa
- Volume V_g= 8.6 cm^3 = 8.6 * 10^{-6} m^3
- Temperature T= 34 ^oC = 34 + 273.15 = 307.15 k
- Gas constant R= 8.314 j/(mol . k)

$$n_g = \frac{(102.5 \ * 10^5) * (8.6 \ * \ 10^{-6} \ )}{8.314 * 307.15}$$

$$n_g = \frac{881.5}{2553.9} \approx 0.345 \text{ moles}$$

❖ Thus, the number of moles of Novec 7000 gas is approximately 0.345 moles. (1)

$$m = M_n \ * \ n_g * \ 10^{-3}$$

Where:

- Molar mass of Novec 7000 M_n = 200 g/mol

$$m = 200 * \ 0.345 * \ 10^{-3} = 0.069 \text{ g}$$

❖ Thus, the mass of Novec 7000 is 69 mg. (2)

$$V_l = \frac{m}{\rho}$$

Where:

- Density of Novec 7000 ρ = 1400 kg/m^3 = 1.4 g/cm^3

$$V_l = \frac{0.069}{1.4} \approx 0.05 \text{ mL}$$

❖ Thus, the volume of Novec 7000 in the system is approximately 5 µL.
